# Supplementary material for: Functional In Vivo Screening Identifies microRNAs Regulating Metastatic Dissemination of Prostate Cancer Cells to Bone Marrow
Source: Cancers (Basel). 2023 Jul 31;15(15):3892. doi: 10.3390/cancers15153892 (PMC10416931; doi:10.3390/cancers15153892)
Supplement: Supplementary file 1 [file cancers-15-03892-s001.zip › cancers-2466399-supplementary.pdf]

Supplementary Figures

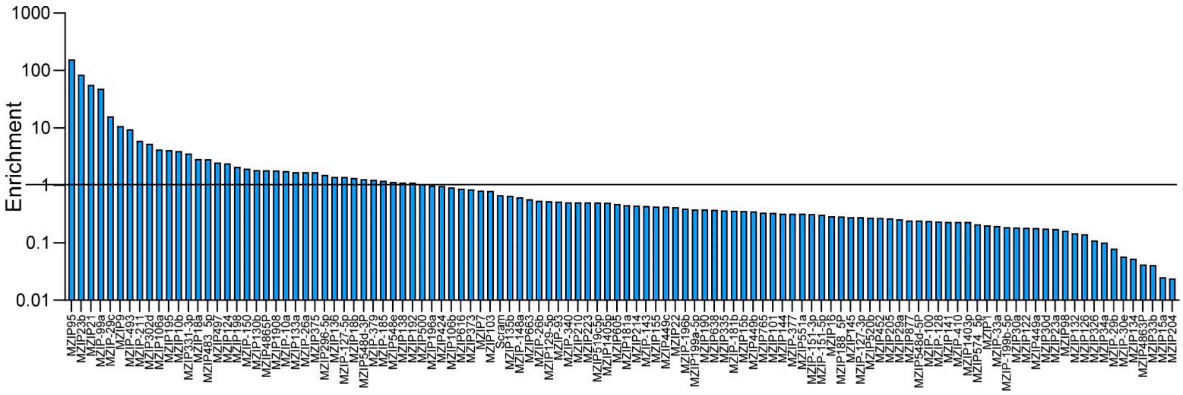

Supplementary Figure S1. Enrichment of miRZIPs in primary tumours. Average fold enrichment of specific anti-miRNA inserts in primary tumours compared to injected pool.

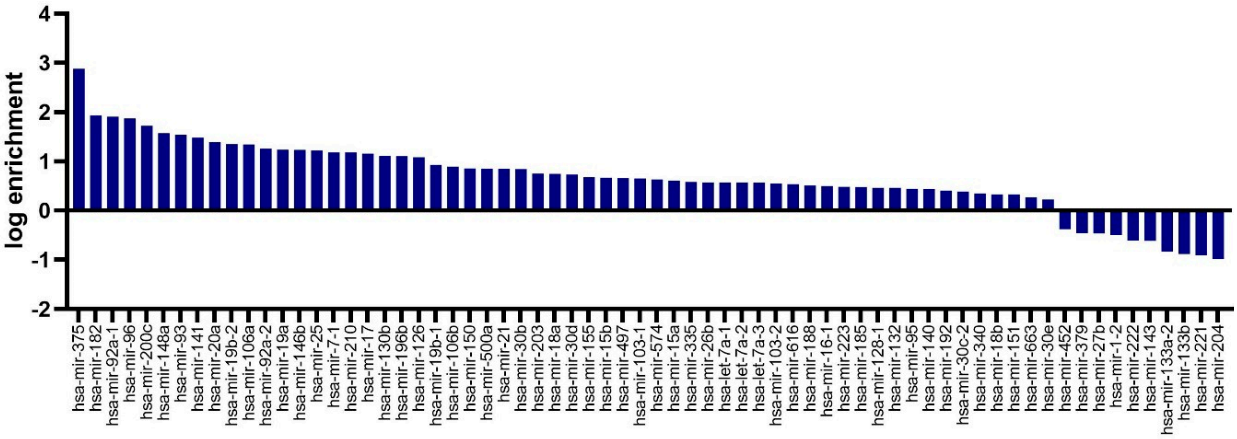

Supplementary Figure S2. Enrichment of miRNAs in primary tumours vs normal. Log fold enrichment of miRNAs targeted by the anti-miRNA in the miRZiP™ lentivirus library in the TCGA PRAD dataset with 52 pairs of tumour and normal prostate tissues.

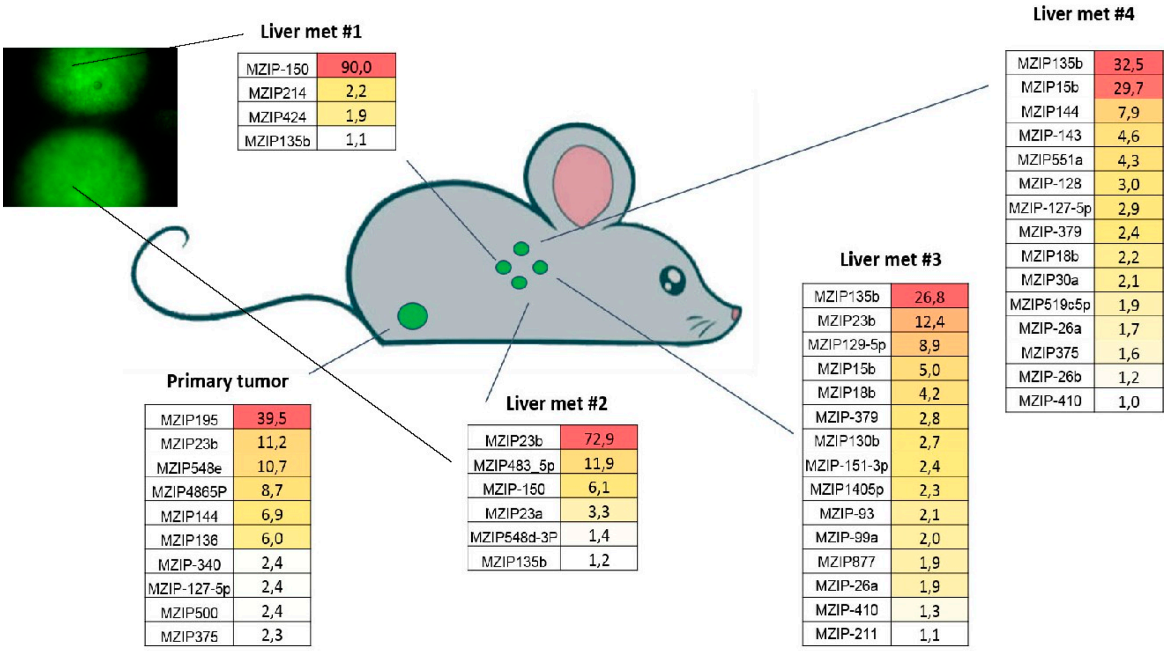

Supplementary Figure S3. Intra-mouse metastatic heterogeneity. Micro-dissection of four different liver metastases from a single mouse gave four different anti-miRNA profiles. Cut-off < 1%.

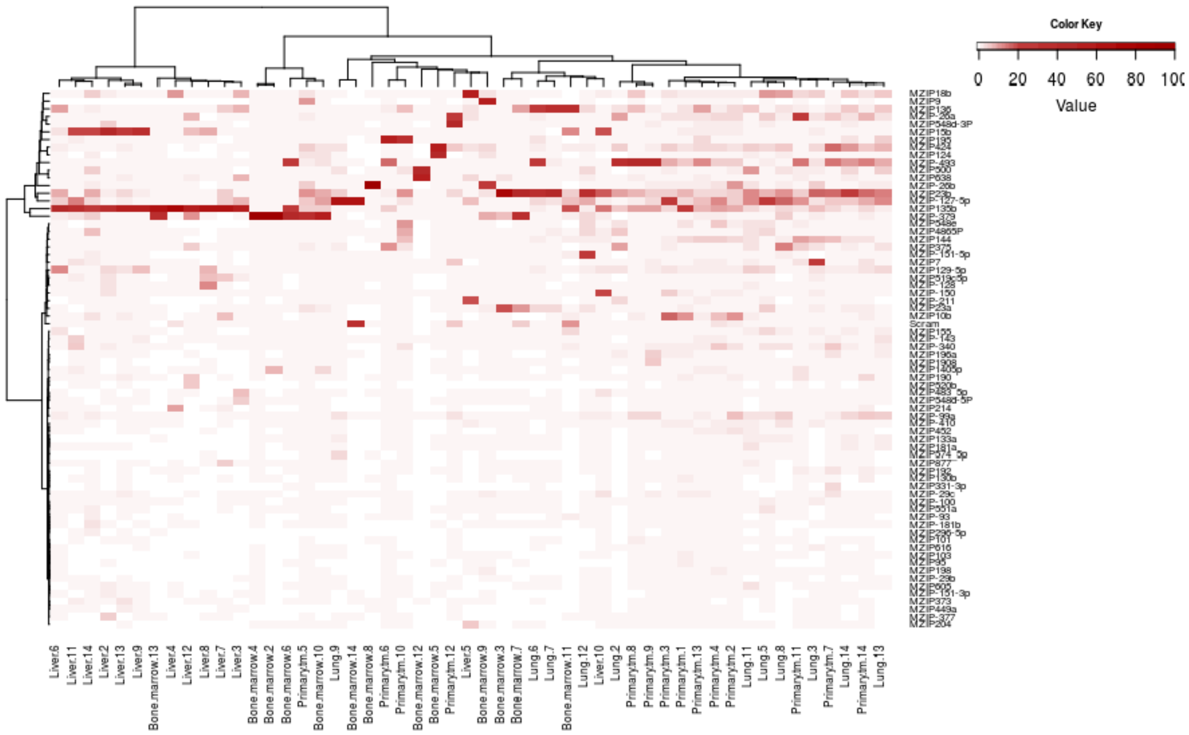

Supplementary Figure S4. Clustering according to sample type not mice. Clustering analysis of all samples collected from orthotopic mouse models including primary tumours, livers, lungs and bone marrow. The analysis was done using Ward's minimum variance method, with the cutoff at 1%.

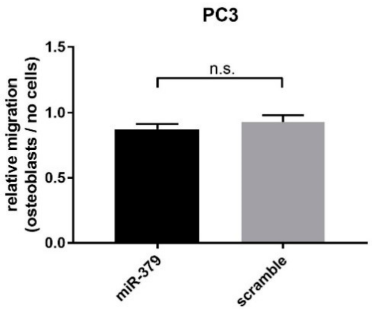

Supplementary Figure S 5. No different of migration in PC3 cell transiently transfected with anti-miR-379 or scrambled towards osteoblasts as attractant.

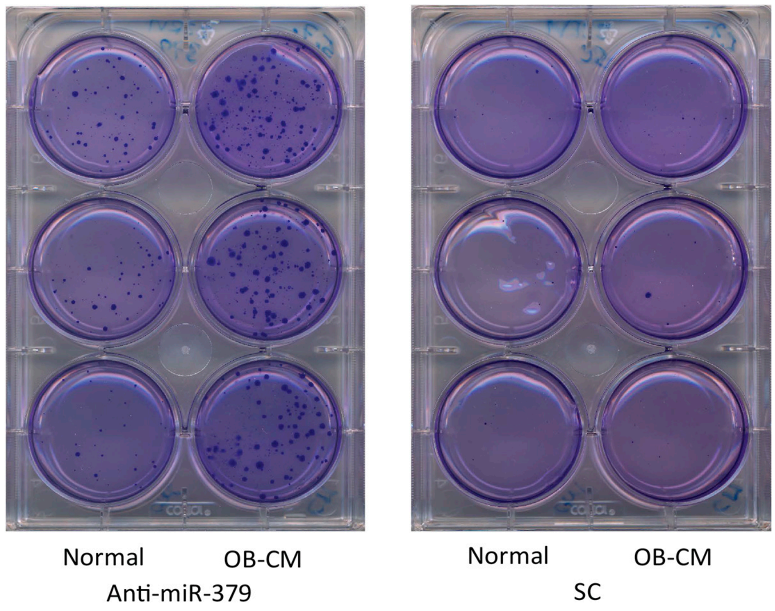

Supplementary Figure S 6. Increased colony formation can be seen in a soft agar colony forming assay for 22Rv1-anti-miR-379 compared to 22Rv1-SC, in OB-CM, but not in normal growth media.

Supplementary Tables

Supplementary Table S1. Primer sequences used for PCR amplification and sequencing.

**Primer**		**Sequence**
miRZip	Forward	5’-TGCATGTCGCTATGTGTTCTGGGA-3’
	Reverse	5´-CTCCCAGGCTCAGATCTGGTCTAA-3´
EXT	Forward	5´-CTGTGAGGGACAGGGGAGT-3´
	Reverse	5´-TTCCCTAGTTAGCCAGAGAGC-3´

Supplementary Table S2. Presence of anti-miR in PC3 cells at injection and in the primary tumours

**LB PC3**		**Primary PC3 tumours**	
	%		average %
MZIP144	9,985317	MZIP-493	13,1978
MZIP135b	9,693607	MZIP195	7,2454
MZIP-127-5p	4,446044	MZIP23b	7,0084
MZIP424	3,846034	MZIP135b	6,3830
MZIP23a	3,547236	MZIP-127-5p	6,2245
MZIP-26b	3,469705	MZIP-26a	5,4732
MZIP-26a	3,205435	MZIP548d-3P	4,1227
MZIP-340	3,202016	MZIP10b	3,7572
MZIP548d-3P	3,189641	MZIP424	3,7532
MZIP129-5p	2,373082	MZIP144	3,2487
MZIP-410	2,316244	MZIP-379	2,7694
MZIP-379	2,205485	MZIP136	2,7025
MZIP190	2,177363	MZIP-99a	1,9751
MZIP452	2,130165	MZIP-26b	1,8945
MZIP136	1,921182	MZIP548e	1,8807
MZIP15b	1,919747	MZIP375	1,8127
MZIP195	1,765186	MZIP18b	1,7158
MZIP-29b	1,650052	MZIP-340	1,6297
MZIP-100	1,634919	MZIP7	1,2687
MZIP548e	1,629085	MZIP129-5p	1,2677
MZIP7	1,560898	Scram	0,9747
MZIP638	1,531294	MZIP196a	0,9570
Scram	1,434847	MZIP4865P	0,9355
MZIP-493	1,389769	MZIP500	0,9231
MZIP548d-5P	1,283295	MZIP-150	0,8370
MZIP1405p	1,28309	MZIP190	0,8288
MZIP18b	1,265975	MZIP-29c	0,8115
MZIP-128	1,177733	MZIP9	0,7971
MZIP204	1,125384	MZIP15b	0,6909
MZIP-143	1,121465	MZIP192	0,6575
MZIP375	1,067794	MZIP1405p	0,6380
MZIP155	1,02618	MZIP23a	0,6214
MZIP196a	0,95797	MZIP452	0,5778
MZIP10b	0,949971	MZIP638	0,5770
MZIP500	0,875289	MZIP-410	0,5378
MZIP877	0,87162	MZIP130b	0,5330
MZIP101	0,768313	MZIP1908	0,5231
MZIP551a	0,702041	MZIP-143	0,4959
MZIP-127-3p	0,639437	MZIP155	0,4404
MZIP519c5p	0,634423	MZIP-100	0,3948
MZIP-151-5p	0,627176	MZIP-211	0,3483
MZIP214	0,5947	MZIP519c5p	0,3189
MZIP192	0,581368	MZIP548d-5P	0,3133

MZIP-93	0,537042	MZIP-93	0,2804
MZIP335	0,53547	MZIP-128	0,2782
MZIP449a	0,513364	MZIP214	0,2651
MZIP4865P	0,508281	MZIP103	0,2587
MZIP-377	0,504772	MZIP124	0,2583
MZIP181a	0,488158	MZIP101	0,2568
MZIP449b	0,459375	MZIP331-3p	0,2292
MZIP-150	0,426739	MZIP551a	0,2266
MZIP-33a	0,363133	MZIP181a	0,2222
MZIP205	0,334555	MZIP877	0,2144
MZIP29a	0,332959	MZIP335	0,1957
MZIP103	0,320151	MZIP133a	0,1935
MZIP605	0,305224	MZIP-151-5p	0,1931
MZIP574_5p	0,295516	MZIP-127-3p	0,1789
MZIP130b	0,286377	MZIP198	0,1752
MZIP98	0,286035	MZIP95	0,1743
MZIP1908	0,285921	MZIP-377	0,1641
MZIP1403p	0,282275	MZIP449b	0,1630
MZIP141	0,237083	MZIP605	0,1450
MZIP1	0,199046	MZIP-29b	0,1314
MZIP-181b	0,169397	MZIP483_5p	0,1308
MZIP30a	0,149159	MZIP138	0,1236
MZIP616	0,138198	MZIP616	0,1211
MZIP-151-3p	0,129742	MZIP449a	0,0933
MZIP145	0,122267	MZIP205	0,0893
MZIP133a	0,113265	MZIP29a	0,0859
MZIP138	0,109163	MZIP106b	0,0824
MZIP210	0,108229	MZIP497	0,0785
MZIP124	0,10595	MZIP-33a	0,0717
MZIP106b	0,08904	MZIP1403p	0,0653
MZIP198	0,083548	MZIP574_5p	0,0622
MZIP23b	0,082887	MZIP-181b	0,0616
MZIP132	0,074067	MZIP141	0,0551
MZIP9	0,073497	MZIP210	0,0549
MZIP449c	0,07336	MZIP98	0,0464
MZIP663	0,071742	MZIP-185	0,0416
MZIP520b	0,069714	MZIP663	0,0414
MZIP34a	0,068483	MZIP-151-3p	0,0413
MZIP331-3p	0,063584	MZIP1	0,0404
MZIP4863P	0,062399	MZIP373	0,0380
MZIP223	0,060188	MZIP145	0,0343
MZIP-211	0,058296	MZIP-148a	0,0322
MZIP126	0,052462	MZIP449c	0,0314
MZIP-148a	0,051915	MZIP223	0,0305
MZIP-29c	0,051004	MZIP30a	0,0278

MZIP-199b-5p	0,049203	MZIP204	0,0270
MZIP483_5p	0,045739	MZIP520b	0,0191
MZIP373	0,044417	MZIP22	0,0168
MZIP-99a	0,040703	MZIP132	0,0109
MZIP22	0,040042	MZIP16	0,0097
MZIP326	0,034709	MZIP-199b-5p	0,0092
MZIP-185	0,034618	MZIP126	0,0075
MZIP16	0,033296	MZIP34a	0,0070
MZIP497	0,031404	MZIP296-5p	0,0065
MZIP30d	0,028419	MZIP30d	0,0050
MZIP302a	0,025935	MZIP122	0,0044
MZIP122	0,023793	MZIP326	0,0039
MZIP134	0,018847	MZIP765	0,0037
MZIP33b	0,014859	MZIP18a	0,0027
MZIP765	0,011099	MZIP4863P	0,0026
MZIP296-5p	0,004262	MZIP21	0,0026
MZIP15a	0,002302	MZIP30b	0,0025
MZIP199a-3p	0,00139	MZIP302d	0,0024
MZIP95	0,001117	MZIP188_3P	0,0011
MZIP18a	0,000934	MZIP134	0,0010
MZIP188_5P	0,000501	MZIP106a	0,0010
MZIP302d	0,000456	MZIP92a	0,0010
MZIP-10a	0,000342	MZIP675	0,0009
MZIP106a	0,000228	MZIP-10a	0,0006
MZIP-196b	0,000205	MZIP33b	0,0006
MZIP-130a	0,000137	MZIP27b	0,0006
MZIP-30e	0,000114	MZIP302c	0,0003
MZIP20a	4,56E-05	MZIP188_5P	0,0001
MZIP21	4,56E-05	MZIP-196b	0,0001
Bullet34a3	4,56E-05	MZIP15a	0,0001
MZIP17	2,28E-05	MZIP27a	
MZIP199a-5p	2,28E-05	MZIP200a	
MZIP206	2,28E-05	MZIP203	
MZIP367	2,28E-05	MZIP199a-5p	
MZIP371-5p	2,28E-05	MZIP-30e	
MZIP-99b	2,28E-05	MZIP302b	
MZIPlet7a	2,28E-05	Bullet34a3	
redo2963p	2,28E-05	MZIP-130a	
MZIP125a-3p		MZIP17	
MZIP125a-5p		MZIP199a-3p	
MZIP125b		MZIP206	
MZIP133b		MZIP20a	
MZIP146a		MZIP302a	
MZIP-146b_3p		MZIP367	
MZIP-146b_5p		MZIP371-5p	

Supplementary Table S3. Ago2-immunoprecipitation in 22Rv1-anti-miR-379 and 22Rv1-SC grown in normal medium followed by RNA-seq

	Gene_stable_ID	Gene_name	baseMean	log2FoldChange	lfcSE	stat	pvalue	padj
183	ENSG00000139793	MBNL2	133,3749	10,46614	1,223672	8,553063	1,20E-17	6,98E-15
1179	ENSG00000152093	CFC1B	20,46278	7,764163	1,369288	5,670219	1,43E-08	2,48E-06
3168	ENSG00000100979	PLTP	212,274	7,704553	0,999817	7,705962	1,30E-14	5,44E-12
6445	ENSG00000003436	TFPI	115,7615	7,68264	0,828867	9,26884	1,88E-20	1,82E-17
9374	ENSG00000167757	KLK11	18,72523	7,627635	1,416777	5,383795	7,29E-08	1,09E-05
2267	ENSG00000213973	ZNF99	34,4881	7,55011	1,260472	5,989906	2,10E-09	4,33E-07
1596	ENSG00000196109	ZNF676	16,66177	7,466542	1,41117	5,291031	1,22E-07	1,68E-05
5472	ENSG00000275778	AC018630.6	15,7923	7,391085	1,457377	5,0715	3,95E-07	4,26E-05
4368	ENSG00000182836	PLCXD3	15,55588	7,367392	1,507693	4,886535	1,03E-06	9,89E-05
10544	ENSG00000166866	MYO1A	15,23688	7,337367	1,445415	5,076304	3,85E-07	4,23E-05
1047	ENSG00000184735	DDX53	14,00745	7,219231	1,465973	4,924531	8,46E-07	8,39E-05
4115	ENSG00000163331	DAPL1	13,67177	7,186214	1,476062	4,868504	1,12E-06	0,000107
1735	ENSG00000172000	ZNF556	12,18047	7,002937	1,497148	4,677517	2,90E-06	0,000236
6544	ENSG00000127903	ZNF835	11,9995	6,991157	1,477775	4,730866	2,24E-06	0,000188
2919	ENSG00000116106	EPHA4	11,96913	6,976722	1,532392	4,552832	5,29E-06	0,00039
11967	ENSG00000123689	G0S2	11,06279	6,875555	1,517779	4,530011	5,90E-06	0,000425
13264	ENSG00000213931	HBE1	10,69538	6,823267	1,672431	4,07985	4,51E-05	0,002126
12407	ENSG00000139117	CPNE8	10,43528	6,789937	1,525035	4,452317	8,49E-06	0,000571
12494	ENSG00000283599	BX276092.9	10,17828	6,747705	1,524978	4,42479	9,65E-06	0,000635
3646	ENSG00000164294	GPX8	18,45359	6,690037	1,377572	4,856397	1,20E-06	0,000112
9119	ENSG00000172238	ATOH1	9,023084	6,585822	1,596125	4,126133	3,69E-05	0,001804
1129	ENSG00000185015	CA13	52,46534	6,574366	0,903061	7,280091	3,34E-13	1,15E-10
1209	ENSG00000160321	ZNF208	8,978449	6,568616	1,572897	4,176126	2,97E-05	0,001495
14564	ENSG00000122176	FMOD	8,880677	6,552994	1,564558	4,188399	2,81E-05	0,001448
8398	ENSG00000135744	AGT	8,594976	6,494884	1,65078	3,934434	8,34E-05	0,00348
1559	ENSG00000137959	IFI44L	8,072791	6,428647	1,688154	3,808092	0,00014	0,005422
13589	ENSG00000054803	CBLN4	23,65591	6,373586	1,211384	5,26141	1,43E-07	1,92E-05
6781	ENSG00000074276	CDHR2	7,108395	6,236867	1,702638	3,663061	0,000249	0,008561
8186	ENSG00000165841	CYP2C19	6,547781	6,121831	1,717196	3,565017	0,000364	0,011231
11031	ENSG00000156113	KCNMA1	6,597426	6,115242	1,734443	3,525768	0,000422	0,012515
3241	ENSG00000189366	ALG1L	23,26371	6,081129	1,183927	5,136406	2,80E-07	3,29E-05
1850	ENSG00000163406	SLC15A2	6,371951	6,072187	1,805397	3,363352	0,00077	0,019649
1125	ENSG00000164879	CA3	6,306376	6,068506	1,705139	3,55895	0,000372	0,011389
13148	ENSG00000167080	B4GALNT2	6,153461	6,035957	1,753017	3,443183	0,000575	0,015946
5637	ENSG00000145934	TENM2	5,962557	5,990182	1,751345	3,420333	0,000625	0,016776
14697	ENSG00000108576	SLC6A4	5,989465	5,985689	1,729594	3,460747	0,000539	0,015193
12213	ENSG00000186458	DEFB132	5,899619	5,972701	1,759614	3,394325	0,000688	0,018143
11245	ENSG00000013297	CLDN11	9,891493	5,793929	1,461071	3,965535	7,32E-05	0,003185
12589	ENSG00000198429	ZNF69	5,151416	5,757698	1,857831	3,099151	0,001941	0,03818
8180	ENSG00000276490	AL583836.1	5,108239	5,752117	1,804719	3,187264	0,001436	0,030688
11390	ENSG00000176920	FUT2	5,075702	5,733876	1,897894	3,021178	0,002518	0,046458

1002	ENSG00000058866	DGKG	4,8891	5,678494	1,88083	3,019143	0,002535	0,046521
8441	ENSG00000112343	TRIM38	35,71206	5,625926	0,933142	6,029012	1,65E-09	3,45E-07
8876	ENSG00000145242	EPHA5	80,47684	5,60998	0,621793	9,022258	1,84E-19	1,45E-16
14414	ENSG00000260903	XKR7	56,52827	5,365176	0,728094	7,3688	1,72E-13	6,41E-11
10510	ENSG00000168939	SPRY3	13,81027	5,190061	1,312558	3,954158	7,68E-05	0,003309
9273	ENSG00000102401	ARMCX3	13,46612	5,159975	1,306395	3,949781	7,82E-05	0,003359
2948	ENSG00000123407	HOXC12	22,17955	5,15874	1,016635	5,07433	3,89E-07	4,24E-05
7104	ENSG00000156959	LHFPL4	8,773301	4,993788	1,457038	3,427356	0,000609	0,016563
14113	ENSG00000186197	EDARADD	57,23044	4,993007	0,726918	6,868737	6,48E-12	1,77E-09
533	ENSG00000139209	SLC38A4	415,4146	4,854914	0,296349	16,38245	2,55E-60	1,14E-56
9460	ENSG00000180938	ZNF572	7,201414	4,718999	1,548865	3,046746	0,002313	0,043467
13067	ENSG00000268089	GABRQ	49,48479	4,587899	0,651772	7,039116	1,93E-12	6,03E-10
5911	ENSG00000152661	GJA1	355,7329	4,527202	0,290902	15,56266	1,31E-54	4,37E-51
2209	ENSG00000167034	NKX3-1	1585,413	4,492652	0,255421	17,58918	2,98E-69	3,99E-65
6911	ENSG00000102362	SYTL4	15,58733	4,468153	1,052866	4,243798	2,20E-05	0,001211
3964	ENSG00000197696	NMB	52,28179	4,387254	0,628172	6,984161	2,87E-12	8,73E-10
7829	ENSG00000158715	SLC45A3	561,9937	4,369654	0,264988	16,49002	4,33E-61	2,90E-57
8173	ENSG00000040731	CDH10	85,59076	4,362138	0,541032	8,062631	7,47E-16	3,45E-13
3079	ENSG00000100867	DHRS2	83,34401	4,360932	1,12748	3,867858	0,00011	0,004352
8127	ENSG00000112818	MEP1A	19,55128	4,353878	0,990132	4,397268	1,10E-05	0,00069
13263	ENSG00000131771	PPP1R1B	27,5182	4,312968	0,899291	4,795962	1,62E-06	0,000142
1269	ENSG00000186998	EMID1	12,22494	4,305494	1,332131	3,232035	0,001229	0,027444
9674	ENSG00000115616	SLC9A2	121,0838	4,298747	0,453462	9,479841	2,55E-21	3,79E-18
12344	ENSG00000184838	PRR16	54,66006	4,293354	0,702078	6,115206	9,64E-10	2,12E-07
2279	ENSG00000128917	DLL4	8,175278	4,200604	1,352293	3,106283	0,001895	0,037705
7027	ENSG00000145555	MYO10	10,82391	4,165844	1,237364	3,36671	0,000761	0,019598
8483	ENSG00000123095	BHLHE41	172,8001	4,140588	0,447131	9,260343	2,04E-20	1,82E-17
5617	ENSG00000046774	MAGEC2	24,41948	4,118748	0,880483	4,67783	2,90E-06	0,000236
426	ENSG00000090104	RGS1	23,6117	4,079261	0,899162	4,536735	5,71E-06	0,000418
9464	ENSG00000262543	SMIM28	11,47506	4,071557	1,177886	3,456667	0,000547	0,015296
3183	ENSG00000197757	HOXC6	22,28304	4,017097	0,942282	4,26316	2,02E-05	0,001154
8226	ENSG00000188375	H3F3C	10,94251	3,983815	1,146789	3,473886	0,000513	0,014591
12303	ENSG00000131037	EPS8L1	13,30027	3,718739	1,146271	3,244205	0,001178	0,026608
8210	ENSG00000132855	ANGPTL3	9,069638	3,717334	1,241702	2,99374	0,002756	0,049424
14490	ENSG00000163485	ADORA1	10,91228	3,631255	1,170189	3,103137	0,001915	0,037948
5344	ENSG00000026950	BTN3A1	21,96597	3,546462	1,047039	3,387133	0,000706	0,018516
10726	ENSG00000131459	GFPT2	19,12633	3,546448	0,835591	4,24424	2,19E-05	0,001211
4299	ENSG00000136383	ALPK3	14,69783	3,531566	0,987328	3,57689	0,000348	0,010935
13356	ENSG00000189056	RELN	64,90758	3,382654	0,489825	6,905846	4,99E-12	1,45E-09
13310	ENSG00000104863	LIN7B	47,67865	3,323299	0,653259	5,087262	3,63E-07	4,06E-05
8786	ENSG00000180745	CLRN3	12,1213	3,291213	1,052906	3,125837	0,001773	0,036044
3883	ENSG00000121005	CRISPLD1	97,85031	3,283081	0,542224	6,054837	1,41E-09	2,99E-07
10307	ENSG00000157510	AFAP1L1	14,52125	3,27824	0,999672	3,279314	0,001041	0,024631
133	ENSG00000134121	CHL1	19,36973	3,221459	0,984544	3,27203	0,001068	0,025053
4875	ENSG00000196208	GREB1	17,05028	3,205975	0,921868	3,477695	0,000506	0,014447

7771	ENSG00000182107	TMEM30B	14,95183	3,201307	0,936709	3,417611	0,000632	0,016893
738	ENSG00000148584	A1CF	30,25651	3,197906	0,717166	4,459086	8,23E-06	0,00056
11530	ENSG00000106004	HOXA5	25,46567	3,161475	0,72741	4,346206	1,39E-05	0,000843
4791	ENSG00000182601	HS3ST4	37,4991	3,155222	0,657648	4,797737	1,60E-06	0,000142
11309	ENSG00000269437	NXF2B	30,11102	3,154196	0,657595	4,796565	1,61E-06	0,000142
12130	ENSG00000184371	CSF1	31,86448	3,116765	0,646909	4,817932	1,45E-06	0,000132
2127	ENSG00000184408	KCND2	14,19055	3,09274	0,937827	3,297774	0,000975	0,023398
11538	ENSG00000106006	HOXA6	19,42477	3,081928	0,833842	3,696055	0,000219	0,007821
14150	ENSG00000063015	SEZ6	25,63209	3,048346	0,73059	4,172442	3,01E-05	0,001509
6355	ENSG00000152217	SETBP1	17,59616	2,984331	0,846581	3,525156	0,000423	0,012517
8941	ENSG00000116833	NR5A2	21,72242	2,951723	0,820499	3,597471	0,000321	0,010474
12385	ENSG00000143469	SYT14	19,8303	2,90186	0,876807	3,309578	0,000934	0,022718
2747	ENSG00000146376	ARHGAP18	22,41405	2,877755	0,866906	3,319572	0,000902	0,022202
13909	ENSG00000129646	QRICH2	47,61997	2,870742	0,648313	4,42802	9,51E-06	0,000631
1698	ENSG00000178171	AMER3	157,0235	2,832341	0,337681	8,387636	4,96E-17	2,56E-14
6449	ENSG00000257704	INAFM1	43,34119	2,830148	0,576539	4,90886	9,16E-07	8,94E-05
2175	ENSG00000113389	NPR3	39,21947	2,756641	0,604496	4,560227	5,11E-06	0,000378
8030	ENSG00000189057	FAM111B	701,4826	2,72655	0,295191	9,236565	2,55E-20	2,13E-17
5357	ENSG00000186310	NAP1L3	26,27444	2,637426	0,701844	3,757854	0,000171	0,006378
3128	ENSG00000163536	SERPINI1	26,62712	2,62679	0,67789	3,874952	0,000107	0,004252
14269	ENSG00000175602	CCDC85B	20,41588	2,622992	0,845957	3,100622	0,001931	0,038102
10586	ENSG00000140443	IGF1R	72,66273	2,607544	0,456018	5,718076	1,08E-08	1,90E-06
10697	ENSG00000084710	EFR3B	41,87302	2,575147	0,655044	3,931259	8,45E-05	0,003516
10284	ENSG00000170667	RASA4B	20,73692	2,568696	0,798269	3,217831	0,001292	0,028529
3428	ENSG00000178573	MAF	110,3967	2,549348	0,369567	6,898207	5,27E-12	1,50E-09
2769	ENSG00000120549	KIAA1217	25,83211	2,500581	0,700829	3,568032	0,00036	0,011154
9112	ENSG00000182263	FIGN	27,88551	2,481228	0,635719	3,903027	9,50E-05	0,003904
12889	ENSG00000099812	MISP	28,19368	2,480694	0,822462	3,016182	0,00256	0,046785
14277	ENSG00000198648	STK39	374,76	2,470197	0,332671	7,425355	1,12E-13	4,43E-11
7956	ENSG00000125170	DOK4	26,42379	2,456596	0,704731	3,485863	0,000491	0,014073
10516	ENSG00000132688	NES	155,167	2,439376	0,420733	5,797921	6,71E-09	1,22E-06
7259	ENSG00000134323	MYCN	56,35248	2,412101	0,500016	4,824051	1,41E-06	0,000129
2662	ENSG00000089199	CHGB	21,33051	2,351663	0,728772	3,226883	0,001251	0,027897
4777	ENSG00000060140	STYK1	29,59752	2,344949	0,674457	3,476794	0,000507	0,014464
9105	ENSG00000115361	ACADL	27,00541	2,332166	0,665138	3,50629	0,000454	0,013205
13778	ENSG00000215397	SCRT2	55,94425	2,33104	0,503951	4,625532	3,74E-06	0,000288
6615	ENSG00000096060	FKBP5	1543,623	2,321169	0,233855	9,925656	3,22E-23	6,16E-20
13115	ENSG00000171345	KRT19	57,87175	2,305372	0,525825	4,384295	1,16E-05	0,000725
9707	ENSG00000185630	PBX1	110,4971	2,292125	0,383729	5,973284	2,33E-09	4,72E-07
7494	ENSG00000114446	IFT57	58,11079	2,289305	0,514118	4,452879	8,47E-06	0,000571
895	ENSG00000126016	AMOT	91,2566	2,27989	0,38584	5,908898	3,44E-09	6,69E-07
3851	ENSG00000171517	LPAR3	281,8455	2,27236	0,261232	8,698634	3,36E-18	2,05E-15
2441	ENSG00000057704	TMCC3	57,22906	2,21498	0,457571	4,840739	1,29E-06	0,00012
3297	ENSG00000165443	PHYHIPL	43,92661	2,205713	0,632396	3,487869	0,000487	0,013997
7940	ENSG00000106789	CORO2A	109,6789	2,186537	0,394164	5,547278	2,90E-08	4,57E-06

14245	ENSG00000112297	CRYBG1	80,56753	2,168064	0,480234	4,514604	6,34E-06	0,000445
11363	ENSG00000165379	LRFN5	38,45419	2,160284	0,561254	3,849034	0,000119	0,004673
5824	ENSG00000196549	MME	167,2089	2,148549	0,370375	5,801004	6,59E-09	1,21E-06
13877	ENSG00000144868	TMEM108	22,69783	2,138419	0,677315	3,157198	0,001593	0,032984
901	ENSG00000102038	SMARCA1	62,57021	2,120874	0,509516	4,162527	3,15E-05	0,001568
12592	ENSG00000178202	KDELC2	27,46522	2,116054	0,703686	3,007098	0,002638	0,047686
5638	ENSG00000182636	NDN	47,01186	2,10541	0,625804	3,364329	0,000767	0,019649
10409	ENSG00000009694	TENM1	53,27985	2,098601	0,494712	4,242063	2,21E-05	0,001211
874	ENSG00000168143	FAM83B	35,91412	2,095326	0,683757	3,064433	0,002181	0,041863
5209	ENSG00000180592	SKIDA1	31,9823	2,094895	0,676082	3,09858	0,001945	0,038197
77	ENSG00000164647	STEAP1	695,1946	2,09459	0,238996	8,764122	1,88E-18	1,20E-15
7184	ENSG00000186001	LRCH3	41,0201	2,087913	0,592775	3,522266	0,000428	0,012604
13162	ENSG00000278139	AL358075.4	133,1237	2,081157	0,498376	4,175875	2,97E-05	0,001495
8189	ENSG00000184226	PCDH9	42,12706	2,072386	0,533803	3,882302	0,000103	0,004175
605	ENSG00000197747	S100A10	358,7318	2,04527	0,244392	8,368824	5,82E-17	2,89E-14
7551	ENSG00000197991	AL592490.1	311,9625	2,040969	0,368252	5,54232	2,98E-08	4,62E-06
6797	ENSG00000137198	GMPR	32,36571	2,027931	0,618502	3,278778	0,001043	0,024634
5708	ENSG00000126803	HSPA2	38,70143	2,000582	0,570254	3,508231	0,000451	0,013166
3818	ENSG00000173272	MZT2A	33,03194	1,999168	0,605046	3,304157	0,000953	0,023037
12445	ENSG00000125398	SOX9	97,23192	1,998591	0,470884	4,244335	2,19E-05	0,001211
5039	ENSG00000164850	GPER1	59,35285	1,98335	0,46735	4,243819	2,20E-05	0,001211
11010	ENSG00000269405	NXF2	87,03834	1,975879	0,5347	3,695307	0,00022	0,007821
13164	ENSG00000187372	PCDHB13	92,05407	1,974875	0,451187	4,377068	1,20E-05	0,000743
7548	ENSG00000280165	PCDH20	321,0616	1,971804	0,376778	5,233328	1,66E-07	2,14E-05
13822	ENSG00000187398	LUZP2	48,75065	1,966595	0,495577	3,968294	7,24E-05	0,00318
1574	ENSG00000103044	HAS3	37,94869	1,954282	0,531697	3,675558	0,000237	0,008302
6426	ENSG00000131171	SH3BGRL	81,32243	1,948677	0,389816	4,998965	5,76E-07	5,94E-05
4872	ENSG00000102290	PCDH11X	32,43172	1,94164	0,61852	3,139172	0,001694	0,034628
8881	ENSG00000148488	ST8SIA6	55,42436	1,935669	0,534967	3,618299	0,000297	0,009886
3534	ENSG00000117525	F3	104,3897	1,930882	0,384002	5,028307	4,95E-07	5,14E-05
7092	ENSG00000116667	C1orf21	60,16224	1,927739	0,548711	3,513212	0,000443	0,01295
5214	ENSG00000182111	ZNF716	141,4972	1,912898	0,373456	5,122152	3,02E-07	3,46E-05
4257	ENSG00000156140	ADAMTS3	51,73776	1,912844	0,458488	4,172073	3,02E-05	0,001509
13972	ENSG00000254979	AP000781.2	29,62233	1,892452	0,596615	3,171979	0,001514	0,031772
8098	ENSG00000026025	VIM	30,4054	1,875383	0,608454	3,082209	0,002055	0,040068
3253	ENSG00000124942	AHNAK	77,90763	1,865124	0,415335	4,490646	7,10E-06	0,00049
8134	ENSG00000169213	RAB3B	226,8712	1,863903	0,389755	4,782244	1,73E-06	0,00015
3300	ENSG00000188112	C6orf132	137,0834	1,854969	0,368464	5,034331	4,80E-07	5,06E-05
2229	ENSG00000090402	SI	31,31998	1,853001	0,610867	3,033394	0,002418	0,045184
8679	ENSG00000138115	CYP2C8	75,78295	1,821903	0,412842	4,413072	1,02E-05	0,000666
5241	ENSG00000144445	KANSL1L	79,08733	1,816722	0,387585	4,687281	2,77E-06	0,000228
6041	ENSG00000135549	PKIB	75,58236	1,814537	0,433027	4,190355	2,79E-05	0,001442
2960	ENSG00000185133	INPP5J	105,4816	1,796349	0,40922	4,389694	1,14E-05	0,000711
3583	ENSG00000185436	IFNLR1	79,90221	1,778714	0,38874	4,575584	4,75E-06	0,000355
7099	ENSG00000113761	ZNF346	44,50355	1,77403	0,518201	3,423439	0,000618	0,016701

12243	ENSG00000185347	TEDC1	43,95929	1,736886	0,551106	3,151634	0,001624	0,033412
34	ENSG00000157214	STEAP2	801,6213	1,736497	0,235866	7,362206	1,81E-13	6,55E-11
3642	ENSG00000134215	VAV3	50,73842	1,725482	0,531061	3,249122	0,001158	0,02633
12553	ENSG00000178235	SLITRK1	66,67774	1,713736	0,419035	4,089723	4,32E-05	0,002066
5091	ENSG00000152952	PLOD2	113,3525	1,70455	0,371694	4,585895	4,52E-06	0,000341
9610	ENSG00000276043	UHRF1	227,5896	1,696552	0,300691	5,642169	1,68E-08	2,85E-06
951	ENSG00000184900	SUMO3	88,12632	1,69451	0,398944	4,247491	2,16E-05	0,001211
13873	ENSG00000085552	IGSF9	227,5842	1,687886	0,353672	4,772465	1,82E-06	0,000156
9041	ENSG00000113361	CDH6	187,1855	1,681088	0,289071	5,815487	6,05E-09	1,12E-06
4675	ENSG00000137801	THBS1	104,9556	1,668463	0,379374	4,397941	1,09E-05	0,00069
13154	ENSG00000117461	PIK3R3	91,20126	1,652806	0,445141	3,712994	0,000205	0,007416
14689	ENSG00000132017	DCAF15	93,53481	1,637205	0,377488	4,3371	1,44E-05	0,000867
1960	ENSG00000273167	AL359736.1	369,0738	1,636099	0,243957	6,706514	1,99E-11	5,14E-09
454	ENSG00000196227	FAM217B	42,902	1,633704	0,502636	3,250269	0,001153	0,02633
6970	ENSG00000160862	AZGP1	324,4284	1,620678	0,260372	6,22447	4,83E-10	1,12E-07
6196	ENSG00000005249	PRKAR2B	160,3904	1,615	0,319276	5,058317	4,23E-07	4,53E-05
1922	ENSG00000182957	SPATA13	87,06863	1,612026	0,382145	4,218363	2,46E-05	0,001319
14518	ENSG00000138162	TACC2	79,70683	1,604841	0,38376	4,181883	2,89E-05	0,001478
908	ENSG00000122952	ZWINT	309,4779	1,562153	0,297544	5,25016	1,52E-07	2,02E-05
12728	ENSG00000185432	METTL7A	885,9759	1,560603	0,217092	7,188669	6,54E-13	2,14E-10
3889	ENSG00000179348	GATA2	149,6172	1,560437	0,312858	4,987685	6,11E-07	6,25E-05
4839	ENSG00000115657	ABCB6	66,22907	1,546524	0,427819	3,614901	0,0003	0,009988
7800	ENSG00000147257	GPC3	91,38127	1,545509	0,47452	3,256997	0,001126	0,026008
8219	ENSG00000131016	AKAP12	108,5317	1,524152	0,397608	3,8333	0,000126	0,004967
12847	ENSG00000079112	CDH17	129,0968	1,522128	0,326907	4,656144	3,22E-06	0,000257
2363	ENSG00000068078	FGFR3	50,15642	1,521516	0,474985	3,203294	0,001359	0,029645
4803	ENSG00000115884	SDC1	107,2219	1,511821	0,379633	3,982325	6,82E-05	0,003017
2238	ENSG00000013810	TACC3	107,6607	1,503408	0,359053	4,187148	2,82E-05	0,00145
2984	ENSG00000104889	RNASEH2A	138,2459	1,502964	0,409592	3,669418	0,000243	0,008416
7575	ENSG00000006210	CX3CL1	204,8481	1,494292	0,352535	4,238701	2,25E-05	0,001224
4248	ENSG00000078018	MAP2	54,67989	1,493982	0,478518	3,122101	0,001796	0,036339
1166	ENSG00000171848	RRM2	443,1898	1,489703	0,279702	5,326035	1,00E-07	1,42E-05
5907	ENSG00000163040	CCDC74A	83,62464	1,488964	0,450599	3,304409	0,000952	0,023037
11876	ENSG00000075702	WDR62	47,17051	1,486331	0,493568	3,011401	0,0026	0,04727
8625	ENSG00000113368	LMNB1	480,9657	1,485825	0,319909	4,644524	3,41E-06	0,000267
2983	ENSG00000284491	AC020934.3	81,78638	1,45254	0,44875	3,236857	0,001209	0,027166
12785	ENSG00000165300	SLITRK5	342,5492	1,452397	0,248887	5,835556	5,36E-09	1,03E-06
4468	ENSG00000138668	HNRNPD	264,5106	1,450392	0,414409	3,499902	0,000465	0,013467
306	ENSG00000135333	EPHA7	432,7317	1,442021	0,235722	6,117474	9,51E-10	2,12E-07
469	ENSG00000213949	ITGA1	120,8022	1,426303	0,323357	4,410918	1,03E-05	0,000669
3582	ENSG00000165891	E2F7	109,94	1,426209	0,394907	3,611504	0,000304	0,010027
1165	ENSG00000284681	AC007240.1	460,9462	1,421311	0,27851	5,103257	3,34E-07	3,79E-05
5171	ENSG00000111670	GNPTAB	69,38726	1,420827	0,431976	3,289131	0,001005	0,023999
8691	ENSG00000180998	GPR137C	148,961	1,418911	0,320183	4,431569	9,35E-06	0,000624
14253	ENSG00000080839	RBL1	70,54804	1,407385	0,419065	3,358396	0,000784	0,019854

4914	ENSG00000177842	ZNF620	81,5848	1,392834	0,417032	3,339874	0,000838	0,02091
14144	ENSG00000074855	ANO8	98,44634	1,385231	0,409401	3,383558	0,000716	0,01865
10301	ENSG00000138604	GLCE	64,2211	1,377608	0,431258	3,194392	0,001401	0,030084
4173	ENSG00000175895	PLEKHF2	178,4545	1,376631	0,311132	4,424587	9,66E-06	0,000635
1834	ENSG00000153179	RASSF3	943,3784	1,375629	0,200234	6,870098	6,42E-12	1,77E-09
2037	ENSG00000065978	YBX1	1413,435	1,370661	0,21724	6,309419	2,80E-10	6,70E-08
2663	ENSG00000138771	SHROOM3	69,98541	1,365347	0,397801	3,432241	0,000599	0,016434
4551	ENSG00000083720	OXCT1	147,4006	1,363817	0,401776	3,394475	0,000688	0,018143
12124	ENSG00000120802	TMPO	392,0502	1,35994	0,319343	4,258561	2,06E-05	0,001168
8388	ENSG00000112146	FBXO9	98,49281	1,355672	0,392183	3,456728	0,000547	0,015296
5597	ENSG00000163545	NUAK2	268,8769	1,335024	0,278715	4,789918	1,67E-06	0,000145
10584	ENSG00000165816	VWA2	86,503	1,321763	0,417689	3,164468	0,001554	0,032388
4453	ENSG00000100836	PABPN1	300,108	1,317238	0,412285	3,194972	0,001398	0,030072
10568	ENSG00000154654	NCAM2	106,5991	1,312902	0,388924	3,375727	0,000736	0,01908
2818	ENSG00000122778	KIAA1549	855,7146	1,310535	0,233042	5,623595	1,87E-08	3,09E-06
4804	ENSG00000070087	PFN2	170,6349	1,310513	0,362953	3,610701	0,000305	0,010027
6878	ENSG00000117054	ACADM	173,188	1,30086	0,355782	3,65634	0,000256	0,008721
6842	ENSG00000182010	RTKN2	98,19748	1,299525	0,409111	3,176462	0,001491	0,031503
1525	ENSG00000171388	APLN	6218,452	1,297157	0,186635	6,95023	3,65E-12	1,09E-09
1430	ENSG00000140534	TICRR	285,0852	1,290292	0,349263	3,694323	0,00022	0,007821
5680	ENSG00000100714	MTHFD1	150,3123	1,288514	0,374406	3,441485	0,000579	0,016014
6376	ENSG00000043143	JADE2	144,7854	1,280436	0,318164	4,024457	5,71E-05	0,002593
6720	ENSG00000147526	TACC1	103,8406	1,268317	0,368048	3,446068	0,000569	0,01581
6080	ENSG00000156802	ATAD2	305,3149	1,268064	0,320308	3,958885	7,53E-05	0,003265
12779	ENSG00000072501	SMC1A	416,5421	1,258787	0,323968	3,88553	0,000102	0,004133
5662	ENSG00000169439	SDC2	230,8293	1,258564	0,287121	4,383387	1,17E-05	0,000725
13819	ENSG00000116962	NID1	238,848	1,251108	0,317481	3,940729	8,12E-05	0,003412
2979	ENSG00000139880	CDH24	158,45	1,235235	0,318447	3,878938	0,000105	0,004208
1420	ENSG00000122126	OCRL	688,7515	1,234424	0,239554	5,153017	2,56E-07	3,04E-05
6208	ENSG00000099624	ATP5D	219,3621	1,234172	0,373766	3,301987	0,00096	0,023132
611	ENSG00000163191	S100A11	1916,462	1,233228	0,264952	4,654534	3,25E-06	0,000257
3199	ENSG00000137203	TFAP2A	151,8862	1,230261	0,301493	4,080562	4,49E-05	0,002126
5298	ENSG00000115457	IGFBP2	296,5188	1,229218	0,273358	4,496725	6,90E-06	0,000482
4469	ENSG00000258643	BCL2L2-PABPN1	415,1561	1,229195	0,306378	4,012018	6,02E-05	0,002716
7656	ENSG00000158813	EDA	125,1817	1,22751	0,332939	3,686895	0,000227	0,008003
13563	ENSG00000100697	DICER1	144,3216	1,217801	0,324833	3,749006	0,000178	0,006584
2419	ENSG00000163703	CRELD1	103,0775	1,215527	0,359229	3,383708	0,000715	0,01865
11397	ENSG00000148835	TAF5	162,4547	1,208551	0,38383	3,148662	0,00164	0,033702
11398	ENSG00000143158	MPC2	622,3595	1,203318	0,269499	4,465023	8,01E-06	0,000547
1935	ENSG00000158270	COLEC12	624,9882	1,19851	0,244204	4,907829	9,21E-07	8,94E-05
12510	ENSG00000136802	LRRC8A	1521,748	1,187921	0,194636	6,10329	1,04E-09	2,25E-07
13921	ENSG00000167536	DHRS13	98,12042	1,187406	0,348224	3,40989	0,00065	0,017275
2171	ENSG00000123080	CDKN2C	138,1864	1,168434	0,305909	3,819549	0,000134	0,005222
10336	ENSG00000147027	TMEM47	178,9825	1,166291	0,295557	3,946082	7,94E-05	0,003387
156	ENSG00000007968	E2F2	286,2784	1,164707	0,252033	4,621243	3,81E-06	0,000292

14259	ENSG00000147862	NFIB	134,3554	1,161757	0,348054	3,337864	0,000844	0,021023
11612	ENSG00000094804	CDC6	127,1165	1,149125	0,322046	3,568205	0,000359	0,011154
958	ENSG00000092470	WDR76	137,3056	1,146458	0,349588	3,279449	0,00104	0,024631
12143	ENSG00000109906	ZBTB16	147,0148	1,143825	0,374166	3,056995	0,002236	0,042545
3827	ENSG00000169760	NLGN1	115,1643	1,1404	0,332511	3,429657	0,000604	0,016496
13546	ENSG00000123643	SLC36A1	135,6339	1,139451	0,322648	3,531565	0,000413	0,012326
6219	ENSG00000101911	PRPS2	101,7565	1,131129	0,374517	3,020236	0,002526	0,046481
14802	ENSG00000105011	ASF1B	137,3843	1,125447	0,310639	3,623005	0,000291	0,009753
13750	ENSG00000160014	CALM3	2158,457	1,119423	0,200483	5,58362	2,36E-08	3,76E-06
4466	ENSG00000215271	HOMEZ	107,8962	1,112267	0,362688	3,06673	0,002164	0,041717
8306	ENSG00000118985	ELL2	242,0922	1,106381	0,266548	4,150779	3,31E-05	0,001638
5118	ENSG00000242265	PEG10	6467,017	1,096477	0,196363	5,583942	2,35E-08	3,76E-06
12392	ENSG00000125968	ID1	131,3099	1,087911	0,356508	3,051579	0,002276	0,042893
2426	ENSG00000175305	CCNE2	312,042	1,085785	0,287617	3,775103	0,00016	0,006036
4552	ENSG00000081803	CADPS2	100,9153	1,085544	0,358788	3,025588	0,002482	0,046045
279	ENSG00000115216	NRBP1	437,6449	1,082578	0,222694	4,86129	1,17E-06	0,00011
3885	ENSG00000164687	FABP5	253,3925	1,074559	0,305087	3,522143	0,000428	0,012604
10911	ENSG00000197157	SND1	145,8778	1,070423	0,303339	3,5288	0,000417	0,0124
9945	ENSG00000064651	SLC12A2	113,9501	1,061442	0,331517	3,201772	0,001366	0,029649
4058	ENSG00000084444	FAM234B	142,7842	1,061177	0,315468	3,363814	0,000769	0,019649
2089	ENSG00000144136	SLC20A1	187,4529	1,05948	0,287663	3,683057	0,00023	0,008103
7645	ENSG00000075624	ACTB	187,9948	1,057723	0,281435	3,758326	0,000171	0,006378
4408	ENSG00000162636	FAM102B	232,6775	1,056142	0,314189	3,361483	0,000775	0,019709
932	ENSG00000177076	ACER2	203,9836	1,048921	0,330712	3,171709	0,001515	0,031772
1452	ENSG00000073111	MCM2	376,1144	1,047259	0,23817	4,397116	1,10E-05	0,00069
12492	ENSG00000258881	AC007040.2	164,0911	1,045141	0,339406	3,079321	0,002075	0,0404
7326	ENSG00000074695	LMAN1	147,9037	1,040586	0,334496	3,110907	0,001865	0,037462
6170	ENSG00000128595	CALU	810,7848	1,029871	0,287276	3,584952	0,000337	0,010703
4680	ENSG00000111602	TIMELESS	263,2414	1,020508	0,249189	4,095316	4,22E-05	0,002032
14183	ENSG00000198668	CALM1	330,7393	1,01818	0,294106	3,461949	0,000536	0,015157
6586	ENSG00000086289	EPDR1	160,16	1,017547	0,327808	3,104092	0,001909	0,037882
6401	ENSG00000135097	MSI1	151,4378	1,01467	0,316919	3,201676	0,001366	0,029649
3979	ENSG00000112118	MCM3	154,7774	1,012365	0,318904	3,174517	0,001501	0,031615
13203	ENSG00000188486	H2AFX	515,6	1,011817	0,233677	4,329992	1,49E-05	0,000892
4792	ENSG00000111666	CHPT1	404,6121	1,011612	0,236058	4,28544	1,82E-05	0,001067
7672	ENSG00000141682	PMAIP1	202,5773	1,00502	0,28032	3,585256	0,000337	0,010703
3383	ENSG00000173905	GOLIM4	303,1911	1,003382	0,334847	2,996536	0,002731	0,049208
11352	ENSG00000085733	CTTN	171,2454	0,997456	0,31156	3,201489	0,001367	0,029649
2447	ENSG00000005469	CROT	505,0198	0,995153	0,279525	3,56016	0,000371	0,011368
9459	ENSG00000165244	ZNF367	802,1191	0,994113	0,257874	3,855035	0,000116	0,004573
6446	ENSG00000150347	ARID5B	421,2709	0,99407	0,219619	4,526349	6,00E-06	0,000428
8664	ENSG00000197451	HNRNPAB	725,5382	0,992475	0,274678	3,613227	0,000302	0,010003
7161	ENSG00000137693	YAP1	127,4521	0,992204	0,309211	3,208826	0,001333	0,029128
9957	ENSG00000127838	PNKD	132,717	0,990726	0,327581	3,024368	0,002492	0,046167
2120	ENSG00000148671	ADIRF	885,3468	0,990093	0,271822	3,642427	0,00027	0,009137

6681	ENSG00000142634	EFHD2	121,8065	0,985986	0,322934	3,053209	0,002264	0,042889
2015	ENSG00000152102	FAM168B	320,6111	0,979174	0,277393	3,52992	0,000416	0,012375
13285	ENSG00000104852	SNRNP70	245,9489	0,976654	0,306794	3,183419	0,001455	0,031049
745	ENSG00000162613	FUBP1	378,0366	0,97184	0,297638	3,265178	0,001094	0,025488
4327	ENSG00000122756	CNTFR	168,7837	0,965257	0,298589	3,232723	0,001226	0,027424
113	ENSG00000175793	SFN	431,2046	0,957569	0,233162	4,106881	4,01E-05	0,001951
14364	ENSG00000182704	TSKU	367,2414	0,952461	0,250898	3,796205	0,000147	0,00564
11146	ENSG00000198785	GRIN3A	535,1053	0,944266	0,217397	4,343521	1,40E-05	0,00085
14515	ENSG00000106462	EZH2	405,7978	0,940513	0,288743	3,257266	0,001125	0,026008
12316	ENSG00000163932	PRKCD	361,5548	0,935171	0,234793	3,982969	6,81E-05	0,003017
14369	ENSG00000172292	CERS6	1483,452	0,930965	0,198467	4,690785	2,72E-06	0,000225
1791	ENSG00000163251	FZD5	638,8036	0,927422	0,232773	3,98424	6,77E-05	0,003013
8635	ENSG00000167749	KLK4	1801,87	0,910002	0,206885	4,398596	1,09E-05	0,00069
2364	ENSG00000176890	TYMS	352,8647	0,908272	0,272621	3,33163	0,000863	0,02142
8346	ENSG00000008394	MGST1	260,7645	0,905141	0,253279	3,573689	0,000352	0,011018
1513	ENSG00000138814	PPP3CA	800,2817	0,903004	0,221741	4,072343	4,65E-05	0,002188
3360	ENSG00000101191	DIDO1	298,0416	0,896437	0,235286	3,809989	0,000139	0,005397
1852	ENSG00000124766	SOX4	1298,646	0,888352	0,203955	4,355631	1,33E-05	0,000812
13324	ENSG00000137727	ARHGAP20	524,8415	0,886643	0,261169	3,394895	0,000687	0,018143
3803	ENSG00000094916	CBX5	568,4889	0,882776	0,271899	3,246704	0,001167	0,02651
342	ENSG00000178252	WDR6	251,9564	0,882773	0,266314	3,314778	0,000917	0,022504
10286	ENSG00000170515	PA2G4	422,5541	0,8817	0,263405	3,347317	0,000816	0,02051
2557	ENSG00000166483	WEE1	417,0986	0,872745	0,282461	3,089786	0,002003	0,039289
4659	ENSG00000111885	MAN1A1	435,4372	0,872305	0,230972	3,776673	0,000159	0,006015
6571	ENSG00000064393	HIPK2	675,1148	0,871212	0,254737	3,420045	0,000626	0,016776
14418	ENSG00000143476	DTL	235,0877	0,859921	0,267151	3,218853	0,001287	0,0285
2513	ENSG00000157240	FZD1	333,3196	0,854602	0,235742	3,62516	0,000289	0,009696
774	ENSG00000116741	RGS2	2781,108	0,838084	0,249678	3,356659	0,000789	0,019911
4288	ENSG00000186575	NF2	745,1033	0,834294	0,23267	3,585745	0,000336	0,010703
2636	ENSG00000102034	ELF4	276,8744	0,83191	0,267848	3,10591	0,001897	0,037705
11084	ENSG00000170779	CDCA4	238,2856	0,829568	0,2607	3,182076	0,001462	0,031144
4306	ENSG00000067113	PLPP1	878,8598	0,821424	0,197607	4,156852	3,23E-05	0,001601
9403	ENSG00000096070	BRPF3	655,1108	0,821179	0,196519	4,178631	2,93E-05	0,001494
11829	ENSG00000213064	SFT2D2	1228,24	0,820671	0,2108	3,893134	9,90E-05	0,00403
1958	ENSG00000111206	FOXM1	352,1832	0,819156	0,235932	3,471999	0,000517	0,014663
9104	ENSG00000110917	MLEC	753,052	0,816091	0,258757	3,153885	0,001611	0,033258
7056	ENSG00000259529	AL136295.5	333,978	0,813322	0,265666	3,061446	0,002203	0,042096
11468	ENSG00000182795	C1orf116	1220,232	0,813059	0,193495	4,201963	2,65E-05	0,001396
4745	ENSG00000065308	TRAM2	335,5319	0,78987	0,261704	3,018179	0,002543	0,046605
7505	ENSG00000135506	OS9	272,0807	0,780192	0,253486	3,077846	0,002085	0,040483
8282	ENSG00000277443	MARCKS	874,922	0,776268	0,189734	4,091355	4,29E-05	0,002059
14342	ENSG00000167900	TK1	323,2442	0,773503	0,250766	3,084561	0,002039	0,039811
1391	ENSG00000187514	PTMA	1809,725	0,761629	0,230786	3,300146	0,000966	0,023243
1560	ENSG00000186767	SPIN4	260,631	0,759656	0,252448	3,00916	0,00262	0,047493
5581	ENSG00000096433	ITPR3	1085,135	0,750087	0,21738	3,450575	0,000559	0,015613

7678	ENSG00000198830	HMGN2	1857,106	0,749382	0,229096	3,271043	0,001072	0,025096
2492	ENSG00000085185	BCORL1	428,4715	0,742835	0,232036	3,201379	0,001368	0,029649
10428	ENSG00000145050	MANF	967,4112	0,741705	0,234491	3,163046	0,001561	0,032429
83	ENSG00000155660	PDIA4	1565,905	0,729131	0,195442	3,730668	0,000191	0,00699
6643	ENSG00000076248	UNG	635,6312	0,724754	0,2123	3,413827	0,000641	0,017095
1645	ENSG00000101412	E2F1	404,0802	0,724018	0,226594	3,195221	0,001397	0,030072
3177	ENSG00000124225	PMEPA1	425,24	0,707775	0,216168	3,274193	0,00106	0,024905
9066	ENSG00000114867	EIF4G1	586,144	0,705625	0,210872	3,346223	0,000819	0,020552
12628	ENSG00000132646	PCNA	1116,931	0,704663	0,207728	3,392245	0,000693	0,018246
5817	ENSG00000005421	PON1	387,687	0,682285	0,223551	3,052028	0,002273	0,042889
6210	ENSG00000169083	AR	1290,523	0,677847	0,186084	3,642688	0,00027	0,009137
194	ENSG00000117318	ID3	360,1539	0,676127	0,224249	3,015072	0,002569	0,046829
13018	ENSG00000188229	TUBB4B	2043,605	0,664782	0,176256	3,77169	0,000162	0,006102
12673	ENSG00000044574	HSPA5	5298,088	0,645839	0,210929	3,061869	0,0022	0,042096
7371	ENSG00000282034	AC106886.5	2184,805	0,61539	0,173557	3,545745	0,000392	0,011893

Supplementary Table S4. Ago2-immunoprecipitation in 22Rv1-anti-miR-379 and 22Rv1-SC grown in normal medium followed by RNA-seq

	Gene_stable_ID	Gene_name	baseMean	log2FoldChange	lfcSE	stat	pvalue	padj
6253	ENSG00000003436	TFPI	71,12244	9,680593	1,32445	7,309139	2,69E-13	1,45E-10
13694	ENSG00000186197	EDARADD	44,4802	8,999401	1,382953	6,507382	7,65E-11	3,03E-08
2219	ENSG00000213973	ZNF99	26,38746	8,232531	1,50202	5,480973	4,23E-08	9,85E-06
12860	ENSG00000131771	PPP1R1B	25,2931	8,186807	1,482282	5,523112	3,33E-08	7,91E-06
7891	ENSG00000112818	MEP1A	19,9467	7,846068	1,583715	4,954218	7,26E-07	0,000137
3090	ENSG00000100979	PLTP	122,4141	7,825687	0,934729	8,372144	5,66E-17	5,17E-14
519	ENSG00000139209	SLC38A4	427,1241	7,634227	0,467037	16,34607	4,64E-60	2,75E-56
12033	ENSG00000139117	CPNE8	16,87029	7,611409	1,704099	4,466529	7,95E-06	0,001061
9021	ENSG00000102401	ARMCX3	14,85838	7,412295	1,738358	4,263962	2,01E-05	0,002149
14127	ENSG00000122176	FMOD	13,682	7,302268	1,698825	4,298424	1,72E-05	0,001909
9208	ENSG00000180938	ZNF572	12,65886	7,167129	2,189423	3,273524	0,001062	0,039416
183	ENSG00000139793	MBNL2	53,15597	7,059404	1,139265	6,196455	5,77E-10	2,02E-07
1160	ENSG00000152093	CFC1B	11,48312	7,028019	1,817929	3,865947	0,000111	0,007964
8869	ENSG00000172238	ATOH1	10,70916	6,960442	1,923179	3,619238	0,000295	0,016169
1116	ENSG00000185015	CA13	31,32465	6,85938	1,390563	4,932807	8,11E-07	0,00015
1036	ENSG00000184735	DDX53	9,716827	6,803845	1,957479	3,475821	0,000509	0,024786
10235	ENSG00000166866	MYO1A	9,38624	6,764654	1,954699	3,460714	0,000539	0,026006
12670	ENSG00000268089	GABRQ	25,9821	6,652812	1,775719	3,746545	0,000179	0,011216
6880	ENSG00000156959	LHFPL4	8,590021	6,634459	1,950635	3,401179	0,000671	0,03018
3551	ENSG00000164294	GPX8	8,341686	6,589402	1,948808	3,381248	0,000722	0,031787
1189	ENSG00000160321	ZNF208	8,295912	6,56441	1,983702	3,309171	0,000936	0,03596
10104	ENSG00000009694	TENM1	25,53709	6,288428	1,410081	4,459621	8,21E-06	0,001071
6698	ENSG00000102362	SYTL4	12,13724	6,163527	1,706104	3,612632	0,000303	0,016435
7973	ENSG00000132855	ANGPTL3	10,6566	5,894454	1,772979	3,324604	0,000885	0,034932
12951	ENSG00000189056	RELN	36,55298	5,842904	1,032231	5,660463	1,51E-08	4,17E-06
13985	ENSG00000260903	XKR7	23,30833	5,652134	1,627357	3,473199	0,000514	0,024928
9411	ENSG00000115616	SLC9A2	129,3306	5,514814	0,575003	9,590938	8,73E-22	1,15E-18
8204	ENSG00000112343	TRIM38	34,56858	5,470617	1,185162	4,615925	3,91E-06	0,000581
2683	ENSG00000146376	ARHGAP18	21,9228	5,457673	1,29458	4,215787	2,49E-05	0,002548
12010	ENSG00000143469	SYT14	19,36261	4,740979	1,226894	3,864214	0,000111	0,007973
2167	ENSG00000167034	NKX3-1	1217,096	4,584093	0,24335	18,83742	3,73E-79	4,43E-75
8645	ENSG00000148488	ST8SIA6	69,10713	4,420963	0,793803	5,569349	2,56E-08	6,33E-06
7937	ENSG00000040731	CDH10	86,64191	4,291861	0,675955	6,349328	2,16E-10	7,78E-08
11969	ENSG00000184838	PRR16	44,25457	4,279668	0,944438	4,531445	5,86E-06	0,000818
5731	ENSG00000152661	GJA1	321,5867	3,993068	0,489323	8,160386	3,34E-16	2,48E-13
7262	ENSG00000114446	IFT57	45,29831	3,934909	0,859381	4,578772	4,68E-06	0,000677
77	ENSG00000164647	STEAP1	876,4938	3,800117	0,234774	16,18631	6,30E-59	2,49E-55
8862	ENSG00000182263	FIGN	23,69688	3,77177	1,039534	3,62833	0,000285	0,015756
7591	ENSG00000158715	SLC45A3	538,424	3,595593	0,294933	12,19124	3,46E-34	1,03E-30
3445	ENSG00000117525	F3	116,1181	3,578758	0,532461	6,721164	1,80E-11	8,23E-09
3342	ENSG00000178573	MAF	52,98179	3,56275	0,885461	4,023612	5,73E-05	0,004793
7792	ENSG00000189057	FAM111B	651,6085	3,561459	0,342483	10,39894	2,51E-25	4,25E-22
8244	ENSG00000123095	BHLHE41	100,3506	3,259625	0,769927	4,233678	2,30E-05	0,002395

5467	ENSG00000182636	NDN	56,0938	3,142512	0,882949	3,559109	0,000372	0,019452
8794	ENSG00000113361	CDH6	109,5206	2,852396	0,42912	6,647078	2,99E-11	1,31E-08
7319	ENSG00000280165	PCDH20	350,2842	2,771274	0,336458	8,236622	1,77E-16	1,40E-13
7322	ENSG00000197991	AL592490.1	341,485	2,751423	0,331623	8,296831	1,07E-16	9,07E-14
2385	ENSG00000057704	TMCC3	60,72493	2,532935	0,648902	3,903417	9,48E-05	0,006995
13852	ENSG00000198648	STK39	423,2639	2,509281	0,291328	8,613245	7,10E-18	7,03E-15
5099	ENSG00000144445	KANSL1L	56,3172	2,496133	0,579205	4,309583	1,64E-05	0,00185
4543	ENSG00000137801	THBS1	155,2596	2,278936	0,418464	5,445956	5,15E-08	1,18E-05
3755	ENSG00000171517	LPAR3	262,6596	2,270491	0,295181	7,691869	1,45E-14	9,06E-12
3730	ENSG00000169760	NLGN1	84,64656	2,259012	0,636221	3,550672	0,000384	0,019926
6409	ENSG00000096060	FKBP5	1008,888	2,154842	0,225308	9,563967	1,13E-21	1,22E-18
1672	ENSG00000178171	AMER3	85,53939	2,086426	0,630052	3,311515	0,000928	0,03596
12066	ENSG00000125398	SOX9	108,8941	2,085039	0,437785	4,762696	1,91E-06	0,000334
30	ENSG00000157214	STEAP2	545,9525	2,039998	0,319204	6,39089	1,65E-10	6,12E-08
594	ENSG00000197747	S100A10	342,6275	2,00626	0,432274	4,641172	3,46E-06	0,000527
12454	ENSG00000079112	CDH17	104,714	1,970989	0,468698	4,205244	2,61E-05	0,002645
3946	ENSG00000084444	FAM234B	133,6366	1,962411	0,419457	4,678455	2,89E-06	0,000452
6753	ENSG00000160862	AZGP1	248,8221	1,907007	0,31567	6,041138	1,53E-09	5,19E-07
5646	ENSG00000196549	MME	126,1632	1,887441	0,451089	4,184185	2,86E-05	0,002786
6512	ENSG00000147526	TACC1	109,7863	1,878457	0,426511	4,404237	1,06E-05	0,001313
5492	ENSG00000169439	SDC2	243,8328	1,747311	0,478202	3,653916	0,000258	0,01462
7898	ENSG00000169213	RAB3B	161,1794	1,740368	0,475098	3,663181	0,000249	0,014343
4423	ENSG00000083720	OXCT1	120,0899	1,692804	0,424835	3,984615	6,76E-05	0,005324
9652	ENSG00000064651	SLC12A2	93,14226	1,666213	0,487215	3,419874	0,000627	0,02929
8111	ENSG00000008394	MGST1	247,8585	1,569946	0,360876	4,350375	1,36E-05	0,001598
6383	ENSG00000086289	EPDR1	182,0248	1,536647	0,3701	4,151978	3,30E-05	0,003106
8046	ENSG00000277443	MARCKS	596,8557	1,448232	0,282767	5,121644	3,03E-07	6,31E-05
2391	ENSG00000005469	CROT	422,0149	1,439914	0,32478	4,433505	9,27E-06	0,001159
1529	ENSG00000186767	SPIN4	205,4277	1,418541	0,374528	3,787546	0,000152	0,010207
303	ENSG00000135333	EPHA7	344,2036	1,3921	0,288829	4,819807	1,44E-06	0,000259
600	ENSG00000163191	S100A11	1240,455	1,330593	0,23839	5,581585	2,38E-08	6,02E-06
11478	ENSG00000213064	SFT2D2	1125,942	1,272244	0,216106	5,887126	3,93E-09	1,17E-06
770	ENSG00000116741	RGS2	2301,152	1,25497	0,227015	5,528136	3,24E-08	7,84E-06
1485	ENSG00000138814	PPP3CA	519,7102	1,250757	0,233256	5,362169	8,22E-08	1,78E-05
13942	ENSG00000172292	CERS6	1331,119	1,224855	0,315515	3,882087	0,000104	0,007499
8395	ENSG00000167749	KLK4	1065,725	1,224585	0,238463	5,13533	2,82E-07	5,97E-05
2750	ENSG00000122778	KIAA1549	661,1372	1,212489	0,343744	3,5273	0,00042	0,021303
4972	ENSG00000242265	PEG10	4379,002	1,16152	0,178629	6,502402	7,90E-11	3,03E-08
1397	ENSG00000122126	OCRL	375,045	1,134282	0,337578	3,360065	0,000779	0,032355
902	ENSG00000122952	ZWINT	291,4961	1,105179	0,31493	3,509291	0,000449	0,022324
12342	ENSG00000185432	METTL7A	755,6806	1,074476	0,268187	4,006443	6,16E-05	0,005083
4658	ENSG00000111666	CHPT1	417,6465	1,069874	0,250211	4,275891	1,90E-05	0,002055
7441	ENSG00000198830	HMGN2	977,3378	1,066272	0,254828	4,184277	2,86E-05	0,002786
4187	ENSG00000067113	PLPP1	926,2904	1,034213	0,233207	4,434747	9,22E-06	0,001159
5641	ENSG00000005421	PON1	398,6208	1,02421	0,273488	3,744987	0,00018	0,011216

1893	ENSG00000158270	COLEC12	529,589	1,009609	0,258228	3,909751	9,24E-05	0,006891
3707	ENSG00000094916	CBX5	468,9138	1,002368	0,249385	4,019363	5,84E-05	0,004846
8385	ENSG00000113368	LMNB1	552,6418	0,989524	0,281065	3,520617	0,000431	0,021482
1800	ENSG00000153179	RASSF3	948,6726	0,825223	0,220164	3,748218	0,000178	0,011216
1992	ENSG00000065978	YBX1	1179,801	0,698894	0,205437	3,401985	0,000669	0,03018
9640	ENSG00000180777	ANKRD30B	17,0198	7,588849	1,840009	4,124355	3,72E-05	0,003449
12769	ENSG00000117472	TSPAN1	15,42557	7,486974	1,701086	4,40129	1,08E-05	0,001317
11719	ENSG00000143184	XCL1	14,54206	7,393124	1,728379	4,277491	1,89E-05	0,002055
1561	ENSG00000164683	HEY1	12,54119	7,146286	2,109288	3,388009	0,000704	0,031195
1123	ENSG00000104267	CA2	11,73903	7,053702	1,992853	3,5395	0,000401	0,020519
818	ENSG00000131781	FMO5	20,99065	6,985363	1,661807	4,203475	2,63E-05	0,002645
8853	ENSG00000176136	MC5R	10,03064	6,856186	1,900074	3,608378	0,000308	0,016631
10675	ENSG00000206549	PRSS50	8,562322	6,600207	1,994477	3,309242	0,000935	0,03596
11094	ENSG00000156298	TSPAN7	7,618839	6,455481	2,01285	3,207134	0,001341	0,048315
4098	ENSG00000185272	RBM11	13,95014	6,362168	1,74197	3,652283	0,00026	0,014628
5239	ENSG00000185261	KIAA0825	13,05377	6,275905	1,69382	3,705178	0,000211	0,012552
2163	ENSG00000213918	DNASE1	13,53557	6,241592	1,747841	3,57103	0,000356	0,018851
1368	ENSG00000173588	CEP83	22,09569	5,862043	1,474347	3,976027	7,01E-05	0,005475
12398	ENSG00000186377	CYP4X1	30,56574	5,362701	1,520819	3,526192	0,000422	0,021303
8655	ENSG00000259030	FPGT-TNNI3K	24,44212	5,34166	1,279855	4,173645	3,00E-05	0,002894
1311	ENSG00000136698	CFC1	119,7602	5,097416	0,728931	6,993002	2,69E-12	1,33E-09
3376	ENSG00000114790	ARHGEF26	21,48277	5,039957	1,438744	3,503025	0,00046	0,022761
8847	ENSG00000166263	STXBP4	18,4807	4,947108	1,463474	3,380387	0,000724	0,031787
6986	ENSG00000172348	RCAN2	39,9507	4,25598	0,90688	4,692993	2,69E-06	0,000432
4990	ENSG00000241399	CD302	33,97768	4,079407	1,124215	3,628671	0,000285	0,015756
641	ENSG00000103534	TMC5	16,70878	4,057408	1,240753	3,270118	0,001075	0,039769
1552	ENSG00000179111	HES7	78,83306	4,033664	0,695261	5,801651	6,57E-09	1,90E-06
2518	ENSG00000276302	AL021997.3	17,67669	4,013129	1,162075	3,453417	0,000554	0,026505
8690	ENSG00000254685	FPGT	24,86006	3,834495	1,08426	3,536509	0,000405	0,020664
3088	ENSG00000205642	VCX3B	46,99879	3,633071	1,021057	3,558148	0,000373	0,019452
3841	ENSG00000089682	RBM41	43,94149	3,552653	0,817304	4,346797	1,38E-05	0,001608
1602	ENSG00000196350	ZNF729	72,01157	3,421883	0,676971	5,054693	4,31E-07	8,39E-05
6203	ENSG00000145569	FAM105A	65,08151	3,407492	0,629401	5,413863	6,17E-08	1,38E-05
8952	ENSG00000164253	WDR41	41,71908	2,890822	0,850984	3,397037	0,000681	0,030525
3297	ENSG00000169860	P2RY1	83,25948	2,880357	0,610399	4,718812	2,37E-06	0,000392
6215	ENSG00000198157	HMGN5	90,73607	2,661225	0,665505	3,998804	6,37E-05	0,005132
5884	ENSG00000102265	TIMP1	83,9389	2,642584	0,648427	4,075375	4,59E-05	0,004011
6671	ENSG00000258289	CHURC1	58,11513	2,593852	0,692992	3,742974	0,000182	0,011248
11025	ENSG00000113369	ARRDC3	78,13943	2,541054	0,562614	4,516516	6,29E-06	0,000868
305	ENSG00000197121	PGAP1	55,79693	2,492875	0,775385	3,215016	0,001304	0,047224
244	ENSG00000153132	CLGN	255,2119	2,46399	0,373444	6,598022	4,17E-11	1,71E-08
3311	ENSG00000164684	ZNF704	58,89251	2,442828	0,613122	3,984247	6,77E-05	0,005324
7255	ENSG00000196604	POTEF	64,98563	2,326337	0,572669	4,062274	4,86E-05	0,004212
1399	ENSG00000213123	TCTEX1D2	89,34193	2,251005	0,546737	4,117165	3,84E-05	0,003531
13781	ENSG00000135747	ZNF670-ZNF695	109,5411	2,124594	0,599995	3,541023	0,000399	0,02049

9470	ENSG00000169570	DTWD2	47,45386	2,094048	0,63575	3,293825	0,000988	0,037497
339	ENSG00000133739	LRRCC1	149,3605	2,084193	0,533236	3,908574	9,28E-05	0,006891
11816	ENSG00000113810	SMC4	119,646	2,042676	0,575619	3,548662	0,000387	0,019991
12552	ENSG00000186871	ERCC6L	78,26302	2,005405	0,600456	3,339804	0,000838	0,033634
2618	ENSG00000184831	APOO	64,96834	1,872036	0,550209	3,402406	0,000668	0,03018
9356	ENSG00000111816	FRK	241,7118	1,849643	0,458803	4,031456	5,54E-05	0,004702
9338	ENSG00000134001	EIF2S1	274,1707	1,712523	0,399222	4,289653	1,79E-05	0,001968
7199	ENSG00000196776	CD47	228,6848	1,708606	0,363849	4,695921	2,65E-06	0,000432
591	ENSG00000179941	BBS10	204,9304	1,634229	0,500009	3,268398	0,001082	0,039887
9869	ENSG00000147592	LACTB2	146,0721	1,58823	0,483271	3,286417	0,001015	0,038237
5084	ENSG00000110315	RNF141	156,7792	1,575879	0,393638	4,003374	6,24E-05	0,005114
1070	ENSG00000105793	GTPBP10	134,1165	1,536299	0,458971	3,34727	0,000816	0,03319
1169	ENSG00000172785	CBWD1	275,2998	1,489492	0,406716	3,662244	0,00025	0,014343
8142	ENSG00000151366	NDUFC2	176,1639	1,400059	0,41941	3,338162	0,000843	0,03372
4067	ENSG00000135317	SNX14	220,0035	1,330357	0,387555	3,432695	0,000598	0,028274
12514	ENSG00000112893	MAN2A1	219,6208	1,327483	0,319589	4,153718	3,27E-05	0,003106
8090	ENSG00000197969	VPS13A	177,3218	1,309205	0,353363	3,704982	0,000211	0,012552
7619	ENSG00000102531	FNDC3A	268,6337	1,308546	0,354874	3,687353	0,000227	0,013255
11959	ENSG00000180776	ZDHHC20	293,3394	1,297501	0,377403	3,437976	0,000586	0,027839
4259	ENSG00000271793	AL589666.1	275,1721	1,287871	0,37019	3,478949	0,000503	0,0246
11116	ENSG00000131711	MAP1B	491,061	1,1983	0,310997	3,853088	0,000117	0,008196
11211	ENSG00000136824	SMC2	406,104	1,17058	0,346785	3,375517	0,000737	0,031915
11545	ENSG00000206527	HACD2	375,6288	1,142093	0,344153	3,318558	0,000905	0,035579
9094	ENSG00000148773	MKI67	278,9055	1,127511	0,314204	3,58847	0,000333	0,017792
4384	ENSG00000142166	IFNAR1	252,2618	1,11847	0,295679	3,782717	0,000155	0,010349
11157	ENSG00000152402	GUCY1A2	1165,221	1,115939	0,223204	4,99964	5,74E-07	0,00011
13185	ENSG00000242028	HYPK	305,6784	1,064855	0,311592	3,417463	0,000632	0,029379
2510	ENSG00000116704	SLC35D1	364,915	1,026592	0,299251	3,430538	0,000602	0,028386
1491	ENSG00000283149	AC068631.2	629,7906	1,001547	0,259537	3,858975	0,000114	0,008097
12145	ENSG00000120738	EGR1	785,334	0,995666	0,228188	4,363364	1,28E-05	0,001521
5537	ENSG00000112697	TMEM30A	578,483	0,995563	0,26802	3,714511	0,000204	0,012268
12122	ENSG00000203667	COX20	400,4576	0,965327	0,284857	3,388813	0,000702	0,031195
2279	ENSG00000138413	IDH1	778,2672	0,957282	0,264299	3,621969	0,000292	0,016074
2777	ENSG00000197147	LRRC8B	382,4701	0,937904	0,278859	3,363364	0,00077	0,032298
11101	ENSG00000143368	SF3B4	588,6688	0,910134	0,282757	3,21878	0,001287	0,046751
690	ENSG00000152291	TGOLN2	807,7847	0,846768	0,252907	3,348135	0,000814	0,03319
1429	ENSG00000090520	DNAJB11	801,9142	0,782291	0,207345	3,772903	0,000161	0,010471
3126	ENSG00000205189	ZBTB10	2590,004	0,659706	0,182556	3,613724	0,000302	0,016435

MZIP-152		MZIP-99b	
MZIP182		MZIPlet7a	
MZIP193b		redo2963p	
MZIP-199b-3p		Bullet34a1	
MZIP19a		Bullet34a2	
MZIP-19b		MZIP125a-3p	
MZIP200a		MZIP125a-5p	
MZIP200c		MZIP125b	
MZIP203		MZIP133b	
MZIP221		MZIP146a	
MZIP222		MZIP-146b_3p	
MZIP224		MZIP-146b_5p	
MZIP24		MZIP-152	
MZIP-25		MZIP182	
MZIP27a		MZIP193b	
MZIP296-3p		MZIP-199b-3p	
MZIP302b		MZIP19a	
MZIP302c		MZIP-19b	
MZIP31		MZIP200b	
MZIP365		MZIP200c	
MZIP371-3p		MZIP221	
MZIP372		MZIP222	
MZIP92a		MZIP224	
MZIP27b		MZIP24	
MZIP31*		MZIP-25	
MZIP33a		MZIP296-3p	
MZIP188_3P		MZIP30c	
MZIP200b		MZIP31	
MZIP483_3p		MZIP31*	
MZIP378		MZIP33a	
MZIP519c3p		MZIP365	
Bullet34a1		MZIP371-3p	
Bullet34a2		MZIP372	
MZIP30b		MZIP378	
MZIP675		MZIP483_3p	
MZIPA2		MZIP4995p	
MZIP4995p		MZIP519c3p	
MZIP30c		MZIP96	
MZIP96		MZIPA2	
